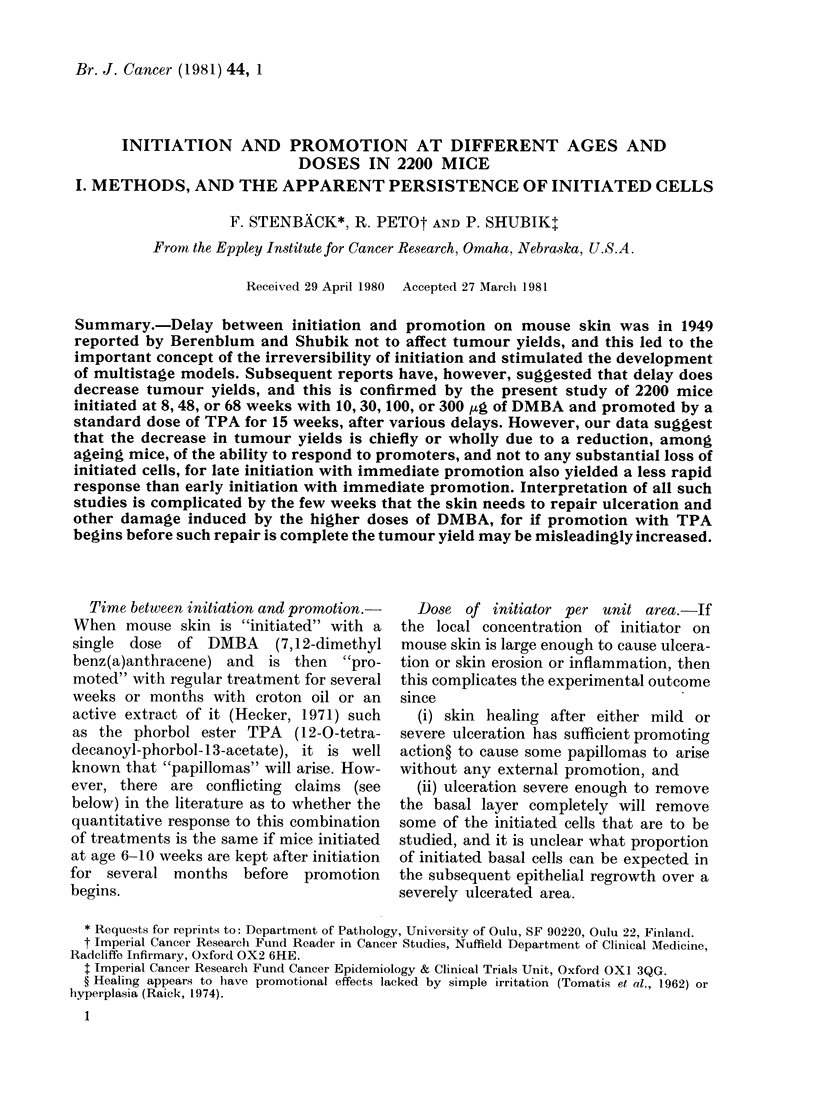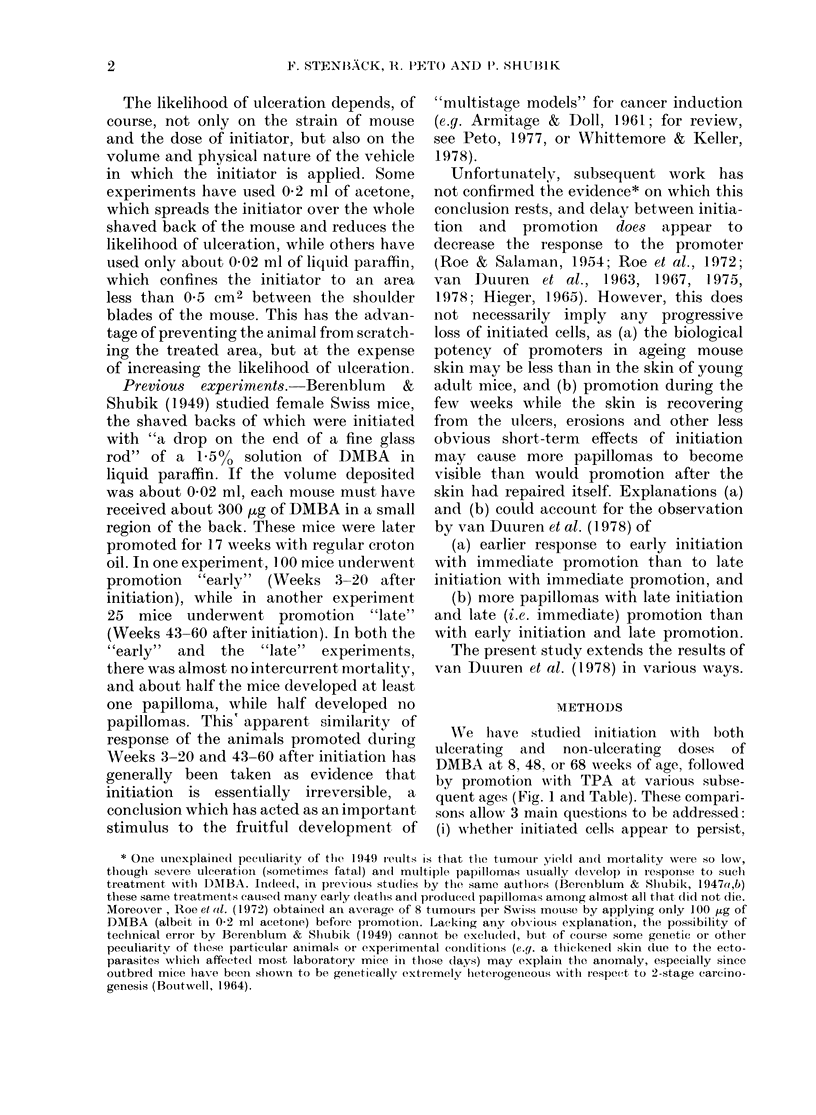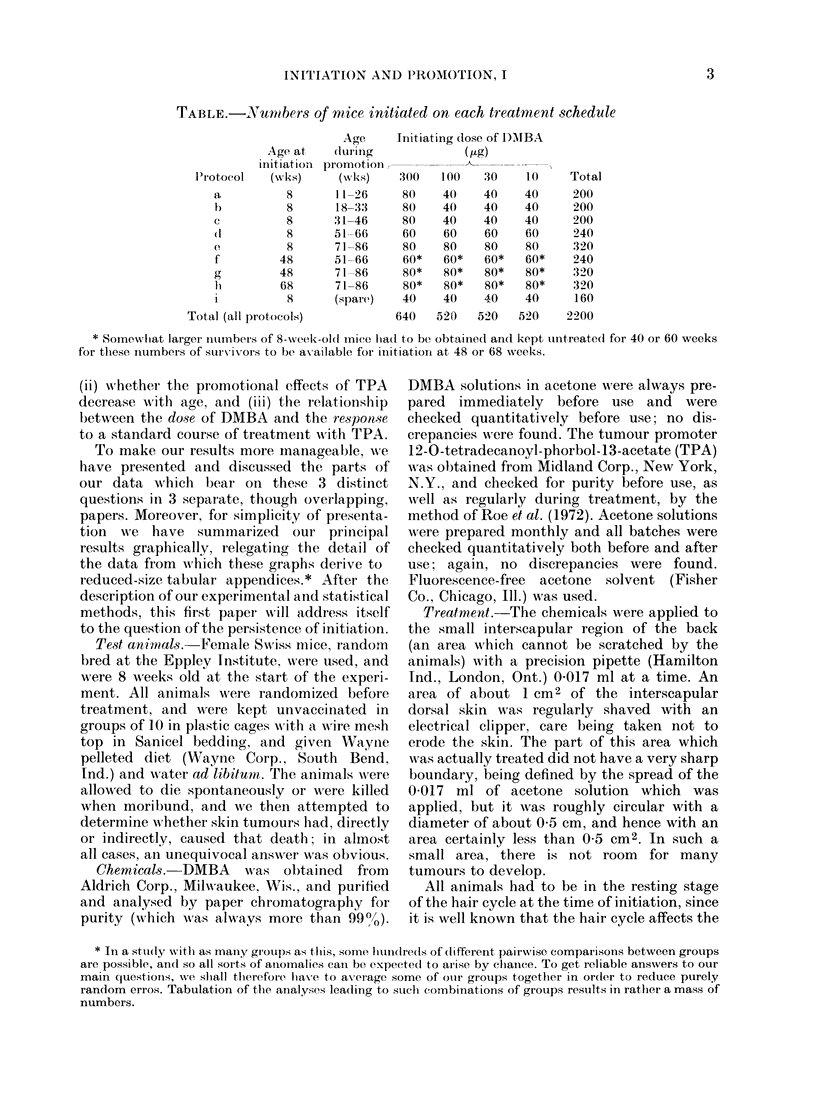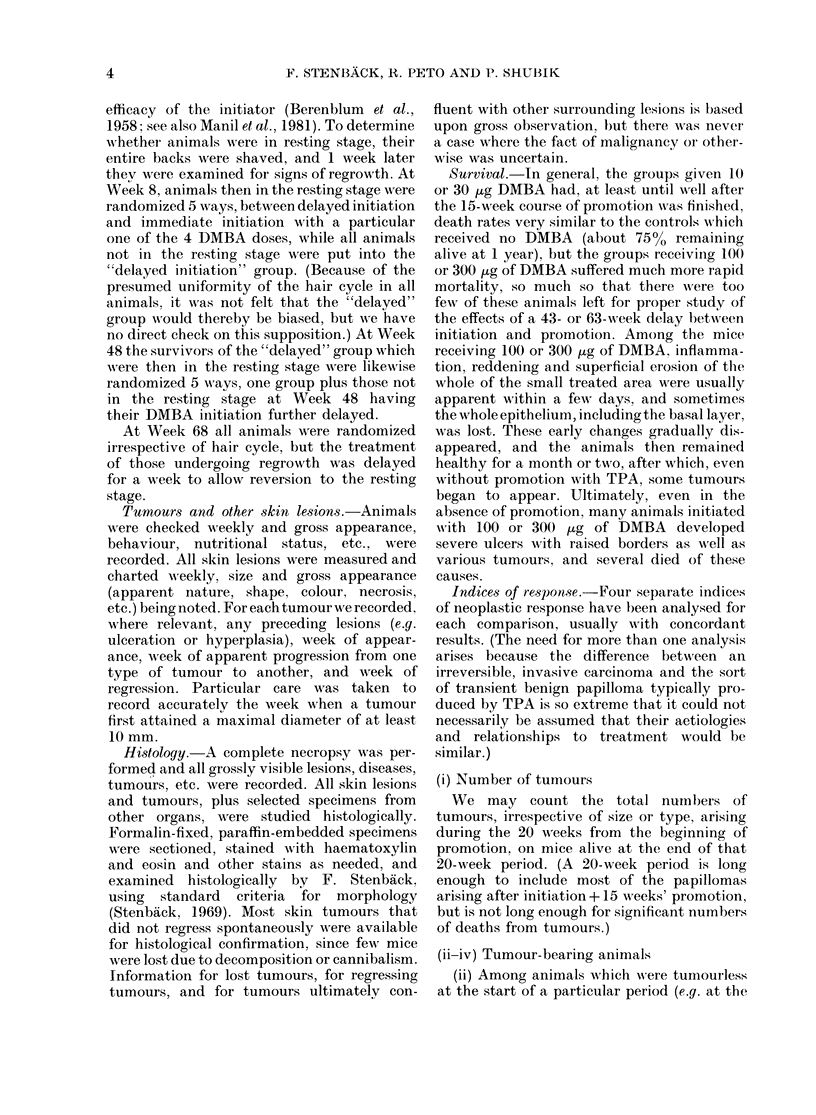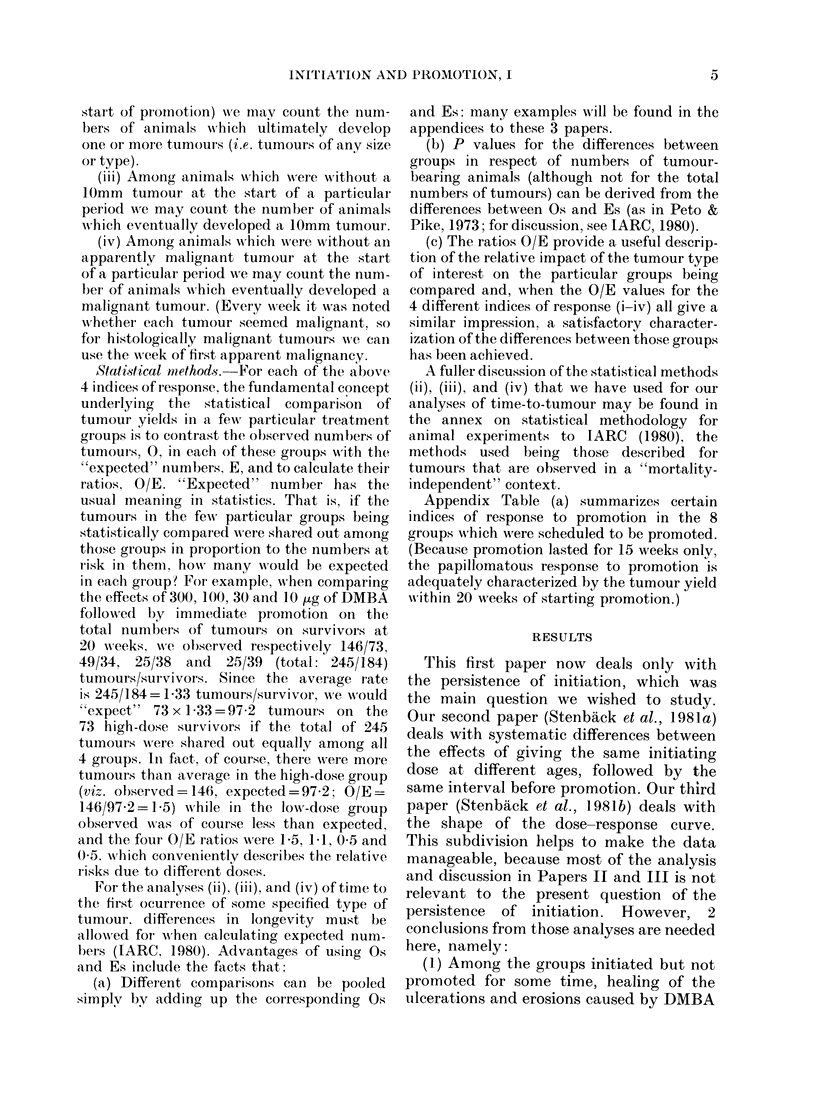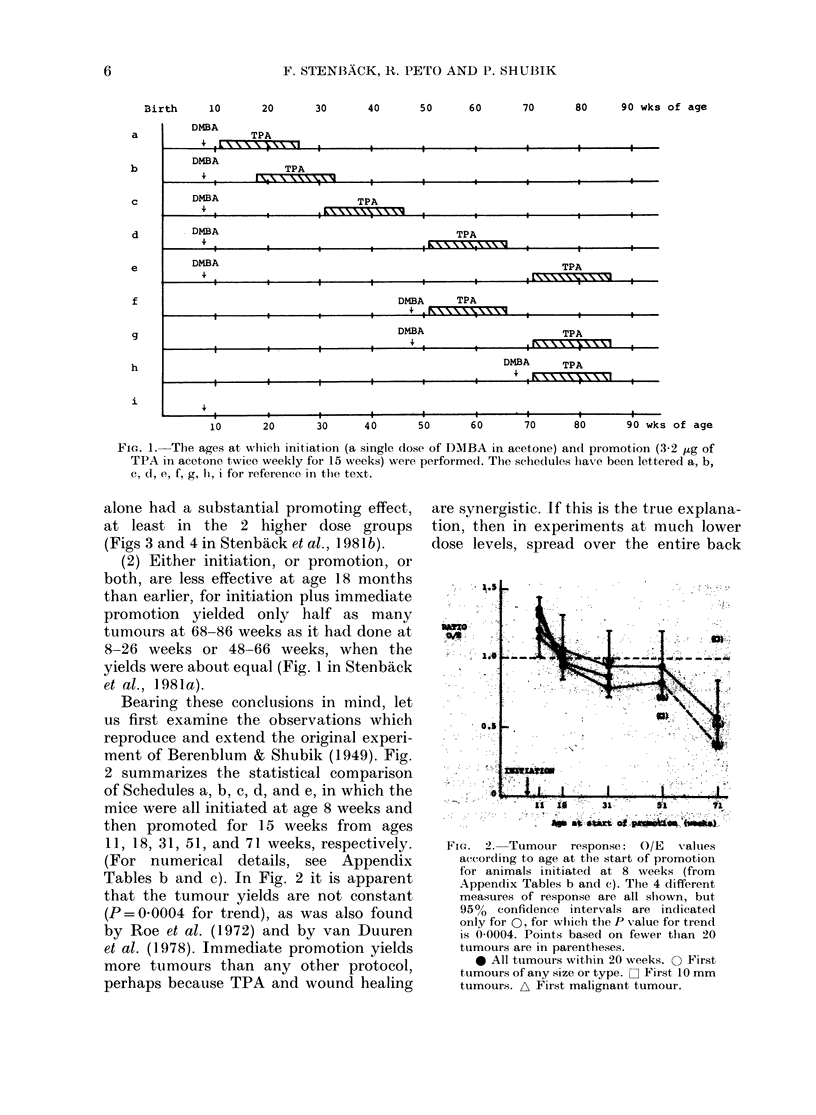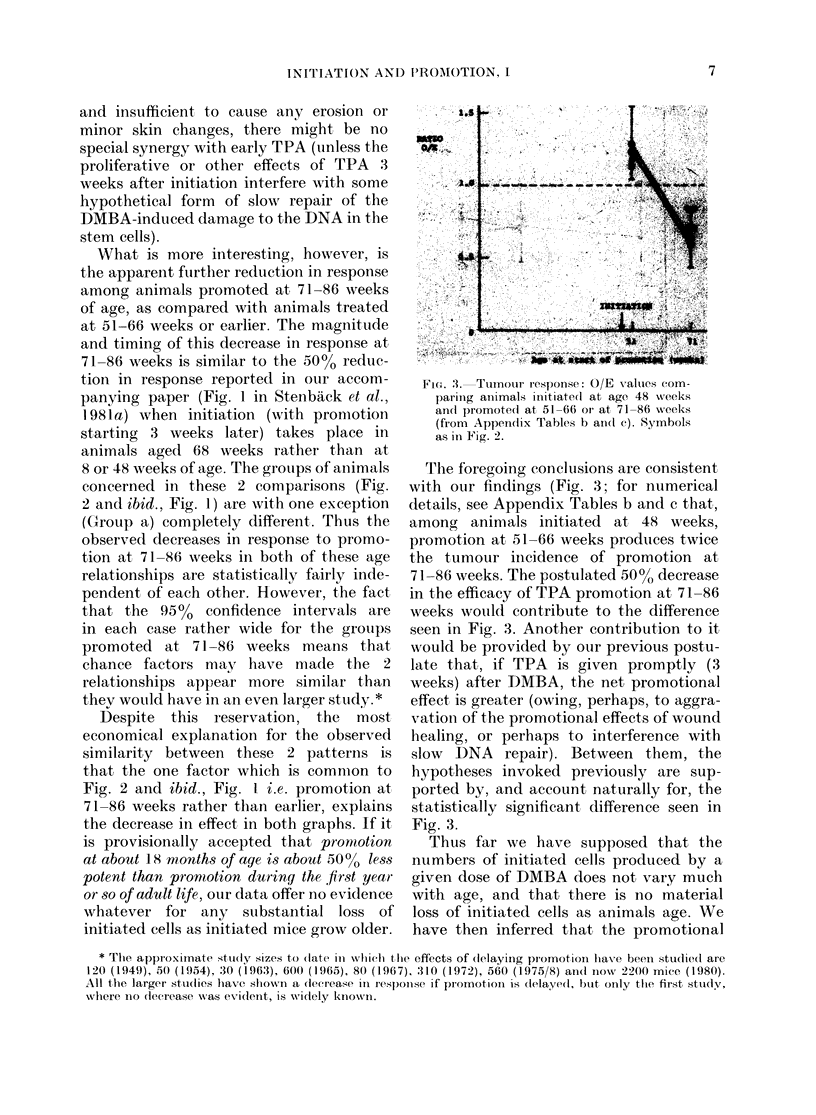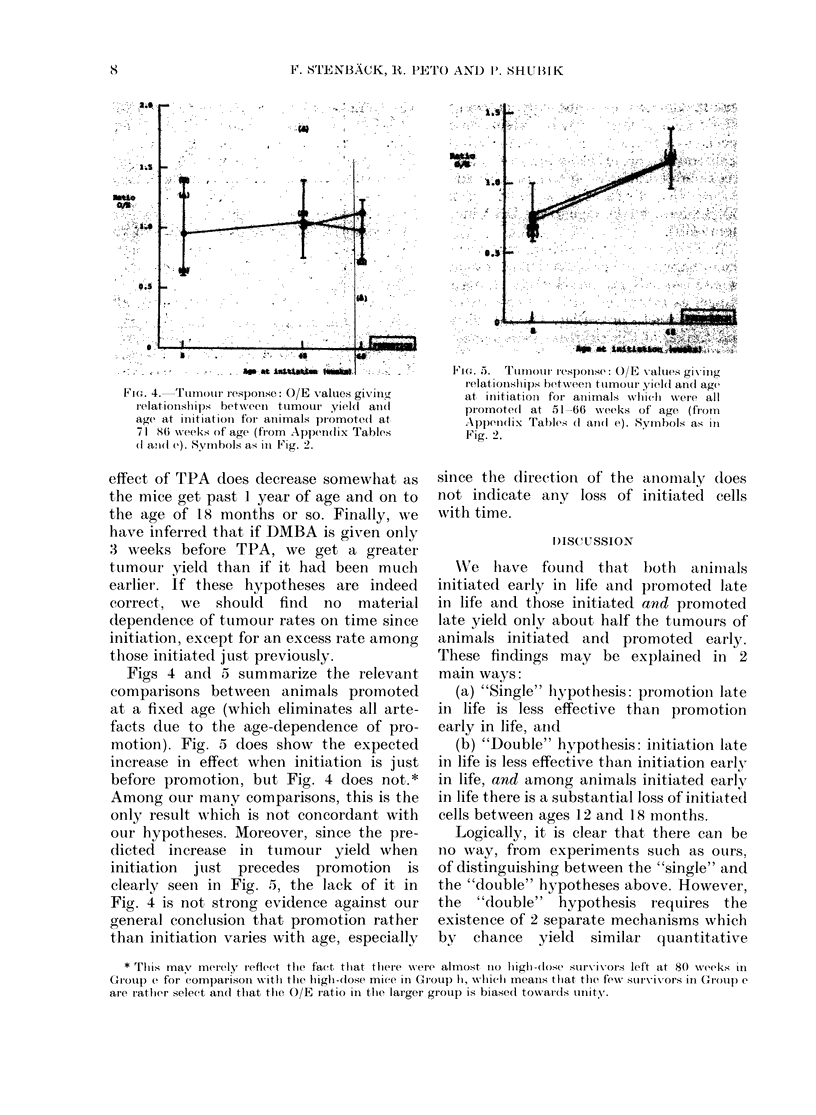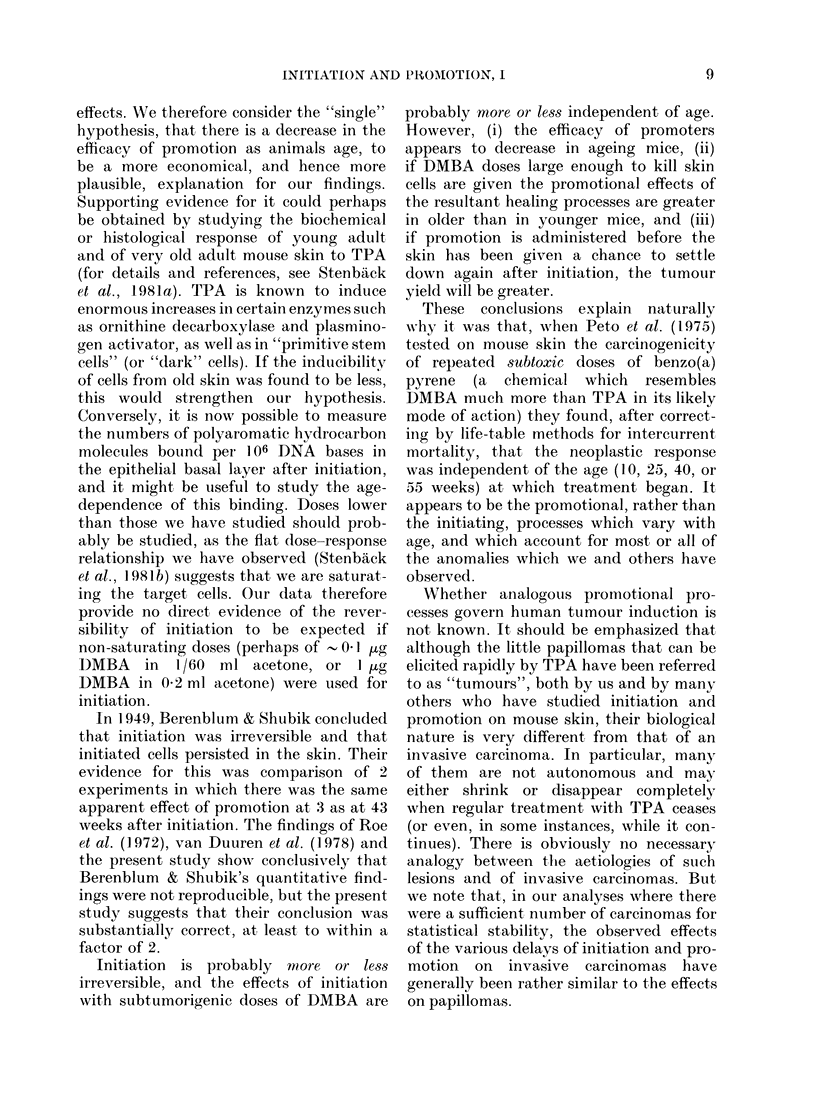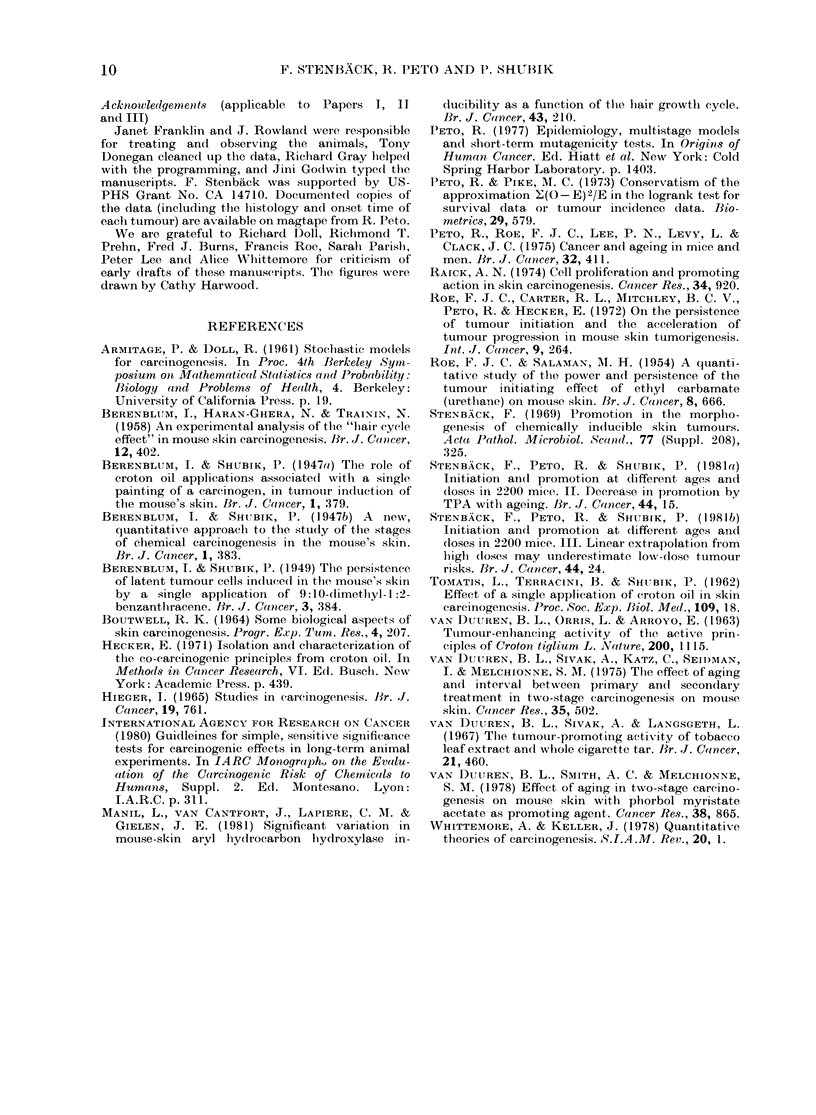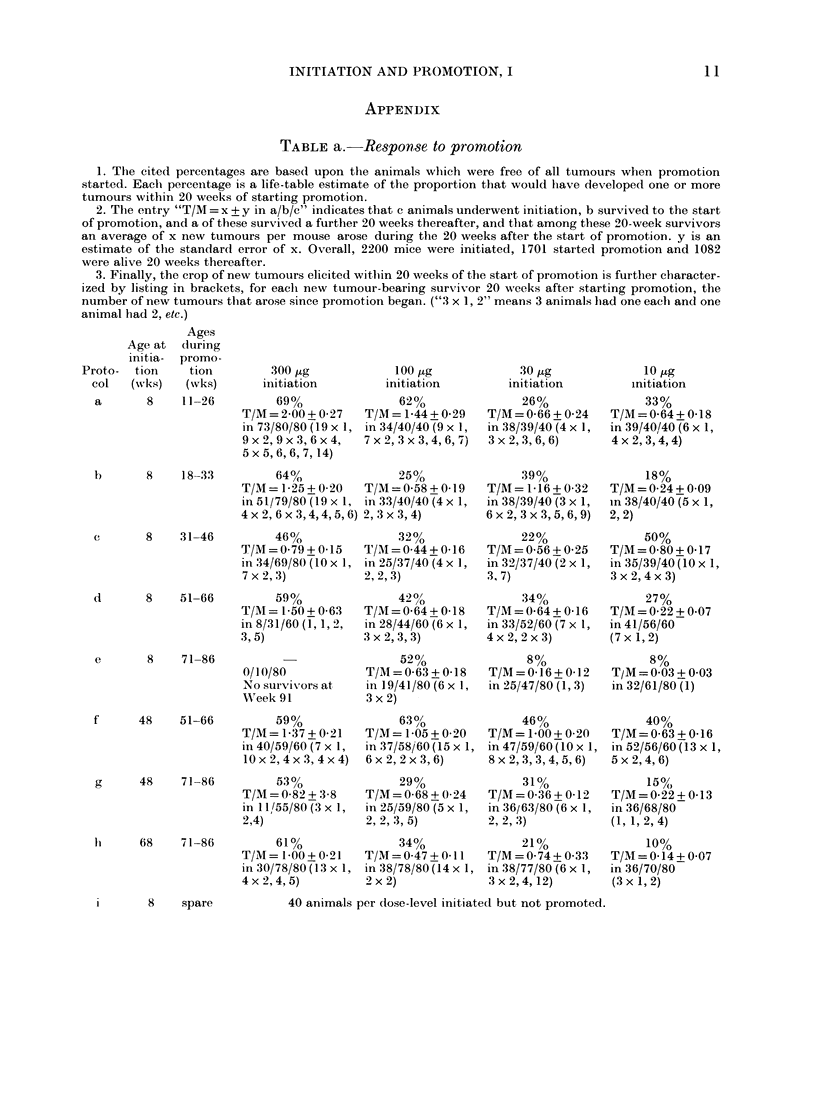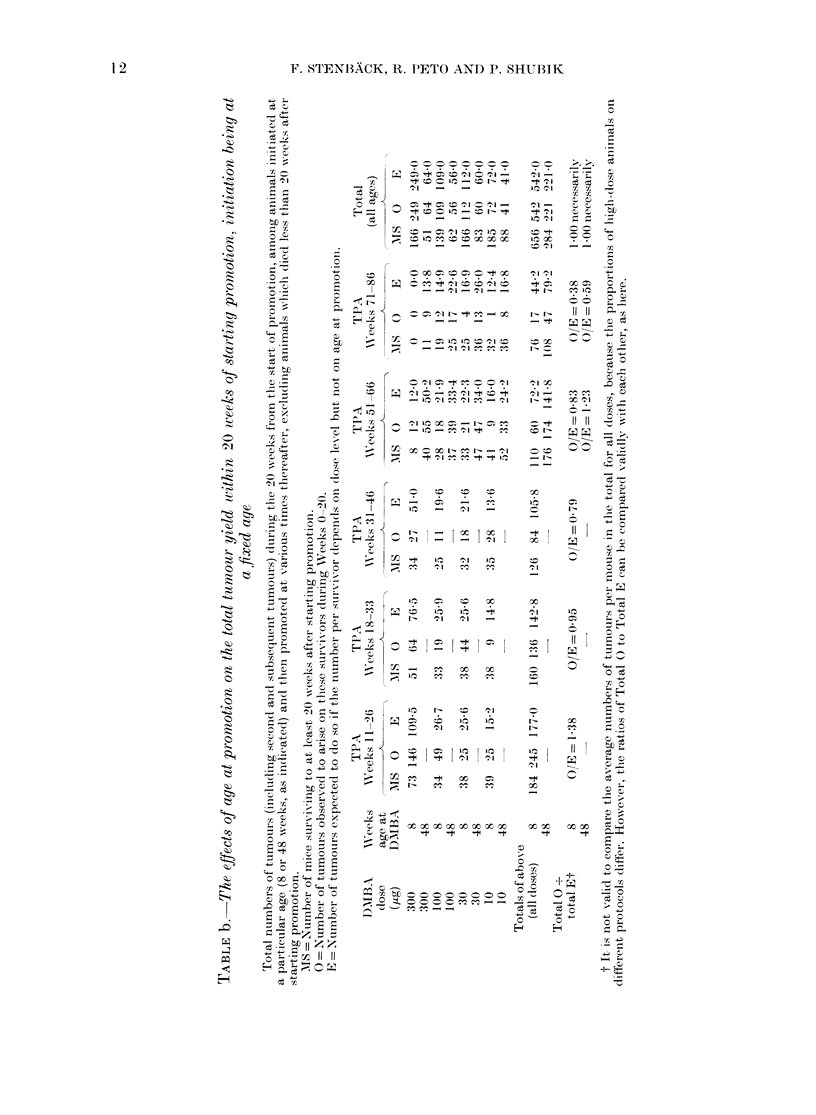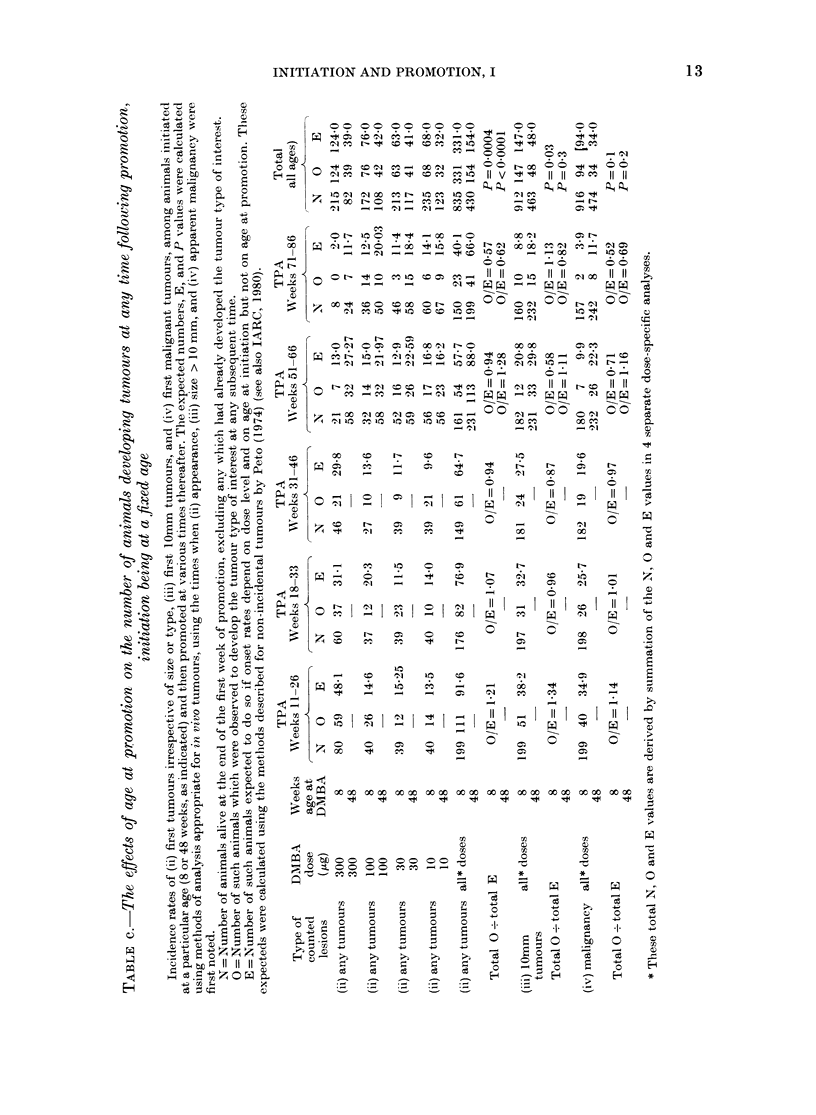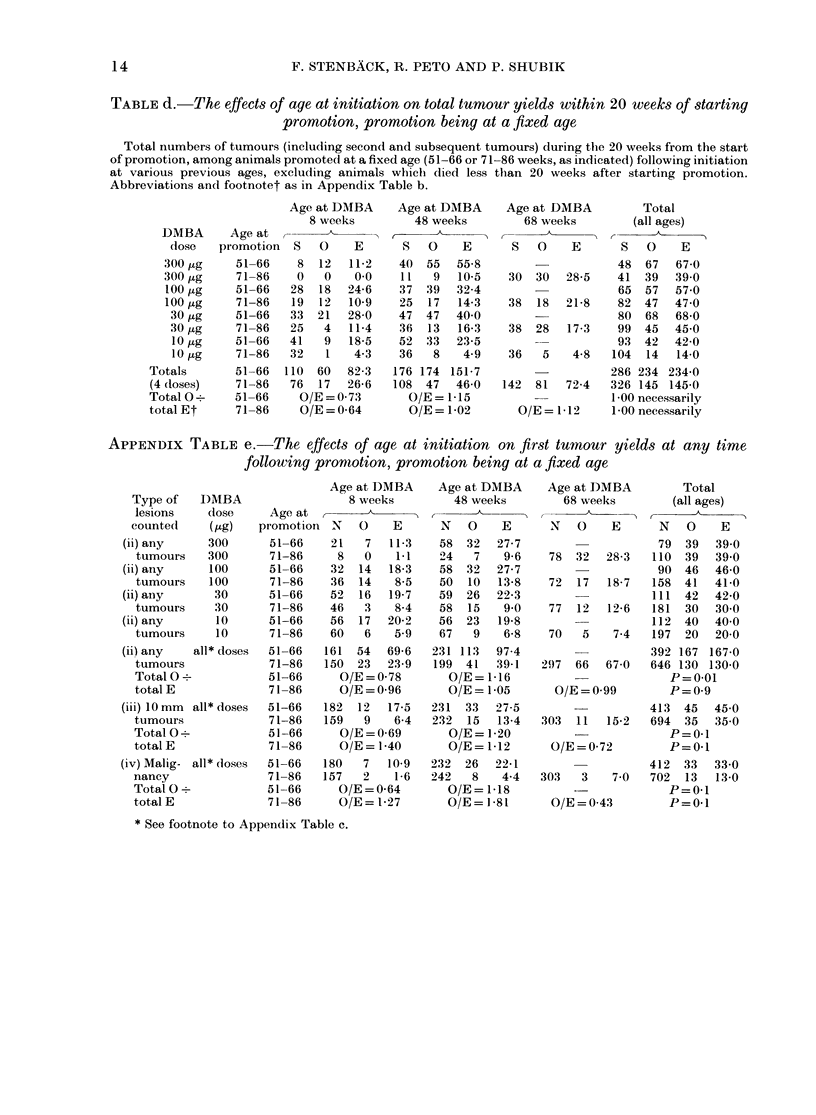# Initiation and promotion at different ages and doses in 2200 mice. I. Methods, and the apparent persistence of initiated cells.

**DOI:** 10.1038/bjc.1981.141

**Published:** 1981-07

**Authors:** F. Stenbäck, R. Peto, P. Shubik

## Abstract

Delay between initiation and promotion on mouse skin was in 1949 reported by Berenblum and Shubik not to affect tumour yields, and this led to the important concept of the irreversibility of initiation and stimulated the development of multistage models. Subsequent reports have, however, suggested that delay does decrease tumour yields, and this is confirmed by the present study of 2200 mice initiated at 8, 48, or 68 weeks with 10, 30, 100, or 300 microgram of DMBA and promoted by a standard dose of TPA for 15 weeks, after various delays. However, our data suggest that the decrease in tumour yields is chiefly or wholly due to a reduction, among ageing mice, of the ability to respond to promoters, and not to any substantial loss of initiated cells, for late initiation with immediate promotion also yielded a less rapid response than early initiation with immediate promotion. Interpretation of all such studies is complicated by the few weeks that the skin needs to repair ulceration and other damage induced by the higher doses of DMBA, for if promotion with TPA begins before such repair is complete the tumour yield may be misleadingly increased.


*Br. J. Cancer* (1981) **44**, 1

### INITIATION AND PROMOTION AT DIFFERENT AGES AND DOSES IN 2200 MICE
### I. METHODS, AND THE APPARENT PERSISTENCE OF INITIATED CELLS

F. STENBÄCK*, R. PETO† AND P. SHUBIK‡

*From the Eppley Institute for Cancer Research, Omaha, Nebraska, U.S.A.*

Received 29 April 1980  Accepted 27 March 1981

**Summary.—Delay between initiation and promotion on mouse skin was in 1949 reported by Berenblum and Shubik not to affect tumour yields, and this led to the important concept of the irreversibility of initiation and stimulated the development of multistage models. Subsequent reports have, however, suggested that delay does decrease tumour yields, and this is confirmed by the present study of 2200 mice initiated at 8, 48, or 68 weeks with 10, 30, 100, or 300 μg of DMBA and promoted by a standard dose of TPA for 15 weeks, after various delays. However, our data suggest that the decrease in tumour yields is chiefly or wholly due to a reduction, among ageing mice, of the ability to respond to promoters, and not to any substantial loss of initiated cells, for late initiation with immediate promotion also yielded a less rapid response than early initiation with immediate promotion. Interpretation of all such studies is complicated by the few weeks that the skin needs to repair ulceration and other damage induced by the higher doses of DMBA, for if promotion with TPA begins before such repair is complete the tumour yield may be misleadingly increased.**

*Time between initiation and promotion.*—When mouse skin is "initiated" with a single dose of DMBA (7,12-dimethyl benz(a)anthracene) and is then "promoted" with regular treatment for several weeks or months with croton oil or an active extract of it (Hecker, 1971) such as the phorbol ester TPA (12-O-tetra-decanoyl-phorbol-13-acetate), it is well known that "papillomas" will arise. However, there are conflicting claims (see below) in the literature as to whether the quantitative response to this combination of treatments is the same if mice initiated at age 6–10 weeks are kept after initiation for several months before promotion begins.

*Dose of initiator per unit area.*—If the local concentration of initiator on mouse skin is large enough to cause ulceration or skin erosion or inflammation, then this complicates the experimental outcome since

(i) skin healing after either mild or severe ulceration has sufficient promoting action§ to cause some papillomas to arise without any external promotion, and

(ii) ulceration severe enough to remove the basal layer completely will remove some of the initiated cells that are to be studied, and it is unclear what proportion of initiated basal cells can be expected in the subsequent epithelial regrowth over a severely ulcerated area.

* Requests for reprints to: Department of Pathology, University of Oulu, SF 90220, Oulu 22, Finland.
† Imperial Cancer Research Fund Reader in Cancer Studies, Nuffield Department of Clinical Medicine, Radcliffe Infirmary, Oxford OX2 6HE.
‡ Imperial Cancer Research Fund Cancer Epidemiology & Clinical Trials Unit, Oxford OX1 3QG.
§ Healing appears to have promotional effects lacked by simple irritation (Tomatis *et al.*, 1962) or hyperplasia (Raick, 1974).

The likelihood of ulceration depends, of course, not only on the strain of mouse and the dose of initiator, but also on the volume and physical nature of the vehicle in which the initiator is applied. Some experiments have used 0·2 ml of acetone, which spreads the initiator over the whole shaved back of the mouse and reduces the likelihood of ulceration, while others have used only about 0·02 ml of liquid paraffin, which confines the initiator to an area less than 0·5 cm$^2$ between the shoulder blades of the mouse. This has the advantage of preventing the animal from scratching the treated area, but at the expense of increasing the likelihood of ulceration.

*Previous experiments.*—Berenblum & Shubik (1949) studied female Swiss mice, the shaved backs of which were initiated with "a drop on the end of a fine glass rod" of a 1·5% solution of DMBA in liquid paraffin. If the volume deposited was about 0·02 ml, each mouse must have received about 300 $\mu$g of DMBA in a small region of the back. These mice were later promoted for 17 weeks with regular croton oil. In one experiment, 100 mice underwent promotion "early" (Weeks 3–20 after initiation), while in another experiment 25 mice underwent promotion "late" (Weeks 43–60 after initiation). In both the "early" and the "late" experiments, there was almost no intercurrent mortality, and about half the mice developed at least one papilloma, while half developed no papillomas. This apparent similarity of response of the animals promoted during Weeks 3–20 and 43–60 after initiation has generally been taken as evidence that initiation is essentially irreversible, a conclusion which has acted as an important stimulus to the fruitful development of "multistage models" for cancer induction (*e.g.* Armitage & Doll, 1961; for review, see Peto, 1977, or Whittemore & Keller, 1978).

Unfortunately, subsequent work has not confirmed the evidence* on which this conclusion rests, and delay between initiation and promotion *does* appear to decrease the response to the promoter (Roe & Salaman, 1954; Roe *et al.*, 1972; van Duuren *et al.*, 1963, 1967, 1975, 1978; Hieger, 1965). However, this does not necessarily imply any progressive loss of initiated cells, as (a) the biological potency of promoters in ageing mouse skin may be less than in the skin of young adult mice, and (b) promotion during the few weeks while the skin is recovering from the ulcers, erosions and other less obvious short-term effects of initiation may cause more papillomas to become visible than would promotion after the skin had repaired itself. Explanations (a) and (b) could account for the observation by van Duuren *et al.* (1978) of

(a) earlier response to early initiation with immediate promotion than to late initiation with immediate promotion, and

(b) more papillomas with late initiation and late (*i.e.* immediate) promotion than with early initiation and late promotion.

The present study extends the results of van Duuren *et al.* (1978) in various ways.

### METHODS

We have studied initiation with both ulcerating and non-ulcerating doses of DMBA at 8, 48, or 68 weeks of age, followed by promotion with TPA at various subsequent ages (Fig. 1 and Table). These comparisons allow 3 main questions to be addressed: (i) whether initiated cells appear to persist,

* One unexplained peculiarity of the 1949 reults is that the tumour yield and mortality were so low, though severe ulceration (sometimes fatal) and multiple papillomas usually develop in response to such treatment with DMBA. Indeed, in previous studies by the same authors (Berenblum & Shubik, 1947*a*,*b*) these same treatments caused many early deaths and produced papillomas among almost all that did not die. Moreover , Roe *et al.* (1972) obtained an average of 8 tumours per Swiss mouse by applying only 100 $\mu$g of DMBA (albeit in 0·2 ml acetone) before promotion. Lacking any obvious explanation, the possibility of technical error by Berenblum & Shubik (1949) cannot be excluded, but of course some genetic or other peculiarity of these particular animals or experimental conditions (*e.g.* a thickened skin due to the ectoparasites which affected most laboratory mice in those days) may explain the anomaly, especially since outbred mice have been shown to be genetically extremely heterogeneous with respect to 2-stage carcinogenesis (Boutwell, 1964).

TABLE.—*Numbers of mice initiated on each treatment schedule*

| Protocol | Age at initiation (wks) | Age during promotion (wks) | Initiating dose of DMBA (μg) 300 | 100 | 30 | 10 | Total |
|---|---|---|---|---|---|---|---|
| a | 8 | 11–26 | 80 | 40 | 40 | 40 | 200 |
| b | 8 | 18–33 | 80 | 40 | 40 | 40 | 200 |
| c | 8 | 31–46 | 80 | 40 | 40 | 40 | 200 |
| d | 8 | 51–66 | 60 | 60 | 60 | 60 | 240 |
| e | 8 | 71–86 | 80 | 80 | 80 | 80 | 320 |
| f | 48 | 51–66 | 60* | 60* | 60* | 60* | 240 |
| g | 48 | 71–86 | 80* | 80* | 80* | 80* | 320 |
| h | 68 | 71–86 | 80* | 80* | 80* | 80* | 320 |
| i | 8 | (spare) | 40 | 40 | 40 | 40 | 160 |
| Total (all protocols) | | | 640 | 520 | 520 | 520 | 2200 |

* Somewhat larger numbers of 8-week-old mice had to be obtained and kept untreated for 40 or 60 weeks for these numbers of survivors to be available for initiation at 48 or 68 weeks.

(ii) whether the promotional effects of TPA decrease with age, and (iii) the relationship between the *dose* of DMBA and the *response* to a standard course of treatment with TPA.

To make our results more manageable, we have presented and discussed the parts of our data which bear on these 3 distinct questions in 3 separate, though overlapping, papers. Moreover, for simplicity of presentation we have summarized our principal results graphically, relegating the detail of the data from which these graphs derive to reduced-size tabular appendices.* After the description of our experimental and statistical methods, this first paper will address itself to the question of the persistence of initiation.

*Test animals.*—Female Swiss mice, random bred at the Eppley Institute, were used, and were 8 weeks old at the start of the experiment. All animals were randomized before treatment, and were kept unvaccinated in groups of 10 in plastic cages with a wire mesh top in Sanicel bedding, and given Wayne pelleted diet (Wayne Corp., South Bend, Ind.) and water *ad libitum*. The animals were allowed to die spontaneously or were killed when moribund, and we then attempted to determine whether skin tumours had, directly or indirectly, caused that death; in almost all cases, an unequivocal answer was obvious.

*Chemicals.*—DMBA was obtained from Aldrich Corp., Milwaukee, Wis., and purified and analysed by paper chromatography for purity (which was always more than 99%). DMBA solutions in acetone were always prepared immediately before use and were checked quantitatively before use; no discrepancies were found. The tumour promoter 12-O-tetradecanoyl-phorbol-13-acetate (TPA) was obtained from Midland Corp., New York, N.Y., and checked for purity before use, as well as regularly during treatment, by the method of Roe *et al.* (1972). Acetone solutions were prepared monthly and all batches were checked quantitatively both before and after use; again, no discrepancies were found. Fluorescence-free acetone solvent (Fisher Co., Chicago, Ill.) was used.

*Treatment.*—The chemicals were applied to the small interscapular region of the back (an area which cannot be scratched by the animals) with a precision pipette (Hamilton Ind., London, Ont.) 0·017 ml at a time. An area of about 1 $cm^2$ of the interscapular dorsal skin was regularly shaved with an electrical clipper, care being taken not to erode the skin. The part of this area which was actually treated did not have a very sharp boundary, being defined by the spread of the 0·017 ml of acetone solution which was applied, but it was roughly circular with a diameter of about 0·5 cm, and hence with an area certainly less than 0·5 $cm^2$. In such a small area, there is not room for many tumours to develop.

All animals had to be in the resting stage of the hair cycle at the time of initiation, since it is well known that the hair cycle affects the

* In a study with as many groups as this, some hundreds of different pairwise comparisons between groups are possible, and so all sorts of anomalies can be expected to arise by chance. To get reliable answers to our main questions, we shall therefore have to average some of our groups together in order to reduce purely random erros. Tabulation of the analyses leading to such combinations of groups results in rather a mass of numbers.

efficacy of the initiator (Berenblum *et al.*, 1958; see also Manil *et al.*, 1981). To determine whether animals were in resting stage, their entire backs were shaved, and 1 week later they were examined for signs of regrowth. At Week 8, animals then in the resting stage were randomized 5 ways, between delayed initiation and immediate initiation with a particular one of the 4 DMBA doses, while all animals not in the resting stage were put into the "delayed initiation" group. (Because of the presumed uniformity of the hair cycle in all animals, it was not felt that the "delayed" group would thereby be biased, but we have no direct check on this supposition.) At Week 48 the survivors of the "delayed" group which were then in the resting stage were likewise randomized 5 ways, one group plus those not in the resting stage at Week 48 having their DMBA initiation further delayed.

At Week 68 all animals were randomized irrespective of hair cycle, but the treatment of those undergoing regrowth was delayed for a week to allow reversion to the resting stage.

*Tumours and other skin lesions.*—Animals were checked weekly and gross appearance, behaviour, nutritional status, etc., were recorded. All skin lesions were measured and charted weekly, size and gross appearance (apparent nature, shape, colour, necrosis, etc.) being noted. For each tumour we recorded, where relevant, any preceding lesions (*e.g.* ulceration or hyperplasia), week of appearance, week of apparent progression from one type of tumour to another, and week of regression. Particular care was taken to record accurately the week when a tumour first attained a maximal diameter of at least 10 mm.

*Histology.*—A complete necropsy was performed and all grossly visible lesions, diseases, tumours, etc. were recorded. All skin lesions and tumours, plus selected specimens from other organs, were studied histologically. Formalin-fixed, paraffin-embedded specimens were sectioned, stained with haematoxylin and eosin and other stains as needed, and examined histologically by F. Stenbäck, using standard criteria for morphology (Stenbäck, 1969). Most skin tumours that did not regress spontaneously were available for histological confirmation, since few mice were lost due to decomposition or cannibalism. Information for lost tumours, for regressing tumours, and for tumours ultimately confluent with other surrounding lesions is based upon gross observation, but there was never a case where the fact of malignancy or otherwise was uncertain.

*Survival.*—In general, the groups given 10 or 30 $\mu$g DMBA had, at least until well after the 15-week course of promotion was finished, death rates very similar to the controls which received no DMBA (about 75% remaining alive at 1 year), but the groups receiving 100 or 300 $\mu$g of DMBA suffered much more rapid mortality, so much so that there were too few of these animals left for proper study of the effects of a 43- or 63-week delay between initiation and promotion. Among the mice receiving 100 or 300 $\mu$g of DMBA, inflammation, reddening and superficial erosion of the whole of the small treated area were usually apparent within a few days, and sometimes the whole epithelium, including the basal layer, was lost. These early changes gradually disappeared, and the animals then remained healthy for a month or two, after which, even without promotion with TPA, some tumours began to appear. Ultimately, even in the absence of promotion, many animals initiated with 100 or 300 $\mu$g of DMBA developed severe ulcers with raised borders as well as various tumours, and several died of these causes.

*Indices of response.*—Four separate indices of neoplastic response have been analysed for each comparison, usually with concordant results. (The need for more than one analysis arises because the difference between an irreversible, invasive carcinoma and the sort of transient benign papilloma typically produced by TPA is so extreme that it could not necessarily be assumed that their aetiologies and relationships to treatment would be similar.)

(i) Number of tumours

We may count the total numbers of tumours, irrespective of size or type, arising during the 20 weeks from the beginning of promotion, on mice alive at the end of that 20-week period. (A 20-week period is long enough to include most of the papillomas arising after initiation + 15 weeks' promotion, but is not long enough for significant numbers of deaths from tumours.)

(ii–iv) Tumour-bearing animals

(ii) Among animals which were tumourless at the start of a particular period (*e.g.* at the start of promotion) we may count the numbers of animals which ultimately develop one or more tumours (*i.e.* tumours of any size or type).

(iii) Among animals which were without a 10mm tumour at the start of a particular period we may count the number of animals which eventually developed a 10mm tumour.

(iv) Among animals which were without an apparently malignant tumour at the start of a particular period we may count the number of animals which eventually developed a malignant tumour. (Every week it was noted whether each tumour seemed malignant, so for histologically malignant tumours we can use the week of first apparent malignancy.

*Statistical methods.*—For each of the above 4 indices of response, the fundamental concept underlying the statistical comparison of tumour yields in a few particular treatment groups is to contrast the observed numbers of tumours, O, in each of these groups with the "expected" numbers, E, and to calculate their ratios, O/E. "Expected" number has the usual meaning in statistics. That is, if the tumours in the few particular groups being statistically compared were shared out among those groups in proportion to the numbers at risk in them, how many would be expected in each group? For example, when comparing the effects of 300, 100, 30 and 10 μg of DMBA followed by immediate promotion on the total numbers of tumours on survivors at 20 weeks, we observed respectively 146/73, 49/34, 25/38 and 25/39 (total: 245/184) tumours/survivors. Since the average rate is 245/184 = 1·33 tumours/survivor, we would "expect" 73 × 1·33 = 97·2 tumours on the 73 high-dose survivors if the total of 245 tumours were shared out equally among all 4 groups. In fact, of course, there were more tumours than average in the high-dose group (*viz.* observed = 146, expected = 97·2; O/E = 146/97·2 = 1·5) while in the low-dose group observed was of course less than expected, and the four O/E ratios were 1·5, 1·1, 0·5 and 0·5, which conveniently describes the relative risks due to different doses.

For the analyses (ii), (iii), and (iv) of time to the first ocurrence of some specified type of tumour, differences in longevity must be allowed for when calculating expected numbers (IARC, 1980). Advantages of using Os and Es include the facts that:

(a) Different comparisons can be pooled simply by adding up the corresponding Os and Es: many examples will be found in the appendices to these 3 papers.

(b) *P* values for the differences between groups in respect of numbers of tumour-bearing animals (although not for the total numbers of tumours) can be derived from the differences between Os and Es (as in Peto & Pike, 1973; for discussion, see IARC, 1980).

(c) The ratios O/E provide a useful description of the relative impact of the tumour type of interest on the particular groups being compared and, when the O/E values for the 4 different indices of response (i–iv) all give a similar impression, a satisfactory characterization of the differences between those groups has been achieved.

A fuller discussion of the statistical methods (ii), (iii), and (iv) that we have used for our analyses of time-to-tumour may be found in the annex on statistical methodology for animal experiments to IARC (1980), the methods used being those described for tumours that are observed in a "mortality-independent" context.

Appendix Table (a) summarizes certain indices of response to promotion in the 8 groups which were scheduled to be promoted. (Because promotion lasted for 15 weeks only, the papillomatous response to promotion is adequately characterized by the tumour yield within 20 weeks of starting promotion.)

### RESULTS

This first paper now deals only with the persistence of initiation, which was the main question we wished to study. Our second paper (Stenbäck *et al.*, 1981*a*) deals with systematic differences between the effects of giving the same initiating dose at different ages, followed by the same interval before promotion. Our third paper (Stenbäck *et al.*, 1981*b*) deals with the shape of the dose–response curve. This subdivision helps to make the data manageable, because most of the analysis and discussion in Papers II and III is not relevant to the present question of the persistence of initiation. However, 2 conclusions from those analyses are needed here, namely:

(1) Among the groups initiated but not promoted for some time, healing of the ulcerations and erosions caused by DMBA

FIG. 1.—The ages at which initiation (a single dose of DMBA in acetone) and promotion (3·2 μg of TPA in acetone twice weekly for 15 weeks) were performed. The schedules have been lettered a, b, c, d, e, f, g, h, i for reference in the text.

alone had a substantial promoting effect, at least in the 2 higher dose groups (Figs 3 and 4 in Stenbäck *et al.*, 1981*b*).

(2) Either initiation, or promotion, or both, are less effective at age 18 months than earlier, for initiation plus immediate promotion yielded only half as many tumours at 68–86 weeks as it had done at 8–26 weeks or 48–66 weeks, when the yields were about equal (Fig. 1 in Stenbäck *et al.*, 1981*a*).

Bearing these conclusions in mind, let us first examine the observations which reproduce and extend the original experiment of Berenblum & Shubik (1949). Fig. 2 summarizes the statistical comparison of Schedules a, b, c, d, and e, in which the mice were all initiated at age 8 weeks and then promoted for 15 weeks from ages 11, 18, 31, 51, and 71 weeks, respectively. (For numerical details, see Appendix Tables b and c). In Fig. 2 it is apparent that the tumour yields are not constant ($P = 0{\cdot}0004$ for trend), as was also found by Roe *et al.* (1972) and by van Duuren *et al.* (1978). Immediate promotion yields more tumours than any other protocol, perhaps because TPA and wound healing are synergistic. If this is the true explanation, then in experiments at much lower dose levels, spread over the entire back

FIG. 2.—Tumour response: O/E values according to age at the start of promotion for animals initiated at 8 weeks (from Appendix Tables b and c). The 4 different measures of response are all shown, but 95% confidence intervals are indicated only for ○, for which the *P* value for trend is 0·0004. Points based on fewer than 20 tumours are in parentheses.

● All tumours within 20 weeks. ○ First tumours of any size or type. □ First 10 mm tumours. △ First malignant tumour.

and insufficient to cause any erosion or minor skin changes, there might be no special synergy with early TPA (unless the proliferative or other effects of TPA 3 weeks after initiation interfere with some hypothetical form of slow repair of the DMBA-induced damage to the DNA in the stem cells).

What is more interesting, however, is the apparent further reduction in response among animals promoted at 71–86 weeks of age, as compared with animals treated at 51–66 weeks or earlier. The magnitude and timing of this decrease in response at 71–86 weeks is similar to the 50% reduction in response reported in our accompanying paper (Fig. 1 in Stenbäck *et al.*, 1981*a*) when initiation (with promotion starting 3 weeks later) takes place in animals aged 68 weeks rather than at 8 or 48 weeks of age. The groups of animals concerned in these 2 comparisons (Fig. 2 and *ibid.*, Fig. 1) are with one exception (Group a) completely different. Thus the observed decreases in response to promotion at 71–86 weeks in both of these age relationships are statistically fairly independent of each other. However, the fact that the 95% confidence intervals are in each case rather wide for the groups promoted at 71–86 weeks means that chance factors may have made the 2 relationships appear more similar than they would have in an even larger study.*

Despite this reservation, the most economical explanation for the observed similarity between these 2 patterns is that the one factor which is common to Fig. 2 and *ibid.*, Fig. 1 *i.e.* promotion at 71–86 weeks rather than earlier, explains the decrease in effect in both graphs. If it is provisionally accepted that *promotion at about* 18 *months of age is about* 50% *less potent than promotion during the first year or so of adult life*, our data offer no evidence whatever for any substantial loss of initiated cells as initiated mice grow older.

FIG. 3.—Tumour response: O/E values comparing animals initiated at age 48 weeks and promoted at 51–66 or at 71–86 weeks (from Appendix Tables b and c). Symbols as in Fig. 2.

The foregoing conclusions are consistent with our findings (Fig. 3; for numerical details, see Appendix Tables b and c that, among animals initiated at 48 weeks, promotion at 51–66 weeks produces twice the tumour incidence of promotion at 71–86 weeks. The postulated 50% decrease in the efficacy of TPA promotion at 71–86 weeks would contribute to the difference seen in Fig. 3. Another contribution to it would be provided by our previous postulate that, if TPA is given promptly (3 weeks) after DMBA, the net promotional effect is greater (owing, perhaps, to aggravation of the promotional effects of wound healing, or perhaps to interference with slow DNA repair). Between them, the hypotheses invoked previously are supported by, and account naturally for, the statistically significant difference seen in Fig. 3.

Thus far we have supposed that the numbers of initiated cells produced by a given dose of DMBA does not vary much with age, and that there is no material loss of initiated cells as animals age. We have then inferred that the promotional

* The approximate study sizes to date in which the effects of delaying promotion have been studied are 120 (1949), 50 (1954), 30 (1963), 600 (1965), 80 (1967), 310 (1972), 560 (1975/8) and now 2200 mice (1980). All the larger studies have shown a decrease in response if promotion is delayed, but only the first study, where no decrease was evident, is widely known.

FIG. 4.—Tumour response: O/E values giving relationships between tumour yield and age at initiation for animals promoted at 71–86 weeks of age (from Appendix Tables d and e). Symbols as in Fig. 2.

FIG. 5. Tumour response: O/E values giving relationships between tumour yield and age at initiation for animals which were all promoted at 51–66 weeks of age (from Appendix Tables d and e). Symbols as in Fig. 2.

effect of TPA does decrease somewhat as the mice get past 1 year of age and on to the age of 18 months or so. Finally, we have inferred that if DMBA is given only 3 weeks before TPA, we get a greater tumour yield than if it had been much earlier. If these hypotheses are indeed correct, we should find no material dependence of tumour rates on time since initiation, except for an excess rate among those initiated just previously.

Figs 4 and 5 summarize the relevant comparisons between animals promoted at a fixed age (which eliminates all artefacts due to the age-dependence of promotion). Fig. 5 does show the expected increase in effect when initiation is just before promotion, but Fig. 4 does not.* Among our many comparisons, this is the only result which is not concordant with our hypotheses. Moreover, since the predicted increase in tumour yield when initiation just precedes promotion is clearly seen in Fig. 5, the lack of it in Fig. 4 is not strong evidence against our general conclusion that promotion rather than initiation varies with age, especially since the direction of the anomaly does not indicate any loss of initiated cells with time.

### DISCUSSION

We have found that both animals initiated early in life and promoted late in life and those initiated *and* promoted late yield only about half the tumours of animals initiated and promoted early. These findings may be explained in 2 main ways:

(a) "Single" hypothesis: promotion late in life is less effective than promotion early in life, and

(b) "Double" hypothesis: initiation late in life is less effective than initiation early in life, *and* among animals initiated early in life there is a substantial loss of initiated cells between ages 12 and 18 months.

Logically, it is clear that there can be no way, from experiments such as ours, of distinguishing between the "single" and the "double" hypotheses above. However, the "double" hypothesis requires the existence of 2 separate mechanisms which by chance yield similar quantitative

* This may merely reflect the fact that there were almost no high-dose survivors left at 80 weeks in Group e for comparison with the high-dose mice in Group h, which means that the few survivors in Group e are rather select and that the O/E ratio in the larger group is biased towards unity.

effects. We therefore consider the "single" hypothesis, that there is a decrease in the efficacy of promotion as animals age, to be a more economical, and hence more plausible, explanation for our findings. Supporting evidence for it could perhaps be obtained by studying the biochemical or histological response of young adult and of very old adult mouse skin to TPA (for details and references, see Stenbäck *et al.*, 1981*a*). TPA is known to induce enormous increases in certain enzymes such as ornithine decarboxylase and plasminogen activator, as well as in "primitive stem cells" (or "dark" cells). If the inducibility of cells from old skin was found to be less, this would strengthen our hypothesis. Conversely, it is now possible to measure the numbers of polyaromatic hydrocarbon molecules bound per $10^6$ DNA bases in the epithelial basal layer after initiation, and it might be useful to study the age-dependence of this binding. Doses lower than those we have studied should probably be studied, as the flat dose–response relationship we have observed (Stenbäck *et al.*, 1981*b*) suggests that we are saturating the target cells. Our data therefore provide no direct evidence of the reversibility of initiation to be expected if non-saturating doses (perhaps of $\sim 0{\cdot}1$ $\mu$g DMBA in 1/60 ml acetone, or 1 $\mu$g DMBA in 0·2 ml acetone) were used for initiation.

In 1949, Berenblum & Shubik concluded that initiation was irreversible and that initiated cells persisted in the skin. Their evidence for this was comparison of 2 experiments in which there was the same apparent effect of promotion at 3 as at 43 weeks after initiation. The findings of Roe *et al.* (1972), van Duuren *et al.* (1978) and the present study show conclusively that Berenblum & Shubik's quantitative findings were not reproducible, but the present study suggests that their conclusion was substantially correct, at least to within a factor of 2.

Initiation is probably *more or less* irreversible, and the effects of initiation with subtumorigenic doses of DMBA are probably *more or less* independent of age. However, (i) the efficacy of promoters appears to decrease in ageing mice, (ii) if DMBA doses large enough to kill skin cells are given the promotional effects of the resultant healing processes are greater in older than in younger mice, and (iii) if promotion is administered before the skin has been given a chance to settle down again after initiation, the tumour yield will be greater.

These conclusions explain naturally why it was that, when Peto *et al.* (1975) tested on mouse skin the carcinogenicity of repeated *subtoxic* doses of benzo(a) pyrene (a chemical which resembles DMBA much more than TPA in its likely mode of action) they found, after correcting by life-table methods for intercurrent mortality, that the neoplastic response was independent of the age (10, 25, 40, or 55 weeks) at which treatment began. It appears to be the promotional, rather than the initiating, processes which vary with age, and which account for most or all of the anomalies which we and others have observed.

Whether analogous promotional processes govern human tumour induction is not known. It should be emphasized that although the little papillomas that can be elicited rapidly by TPA have been referred to as "tumours", both by us and by many others who have studied initiation and promotion on mouse skin, their biological nature is very different from that of an invasive carcinoma. In particular, many of them are not autonomous and may either shrink or disappear completely when regular treatment with TPA ceases (or even, in some instances, while it continues). There is obviously no necessary analogy between the aetiologies of such lesions and of invasive carcinomas. But we note that, in our analyses where there were a sufficient number of carcinomas for statistical stability, the observed effects of the various delays of initiation and promotion on invasive carcinomas have generally been rather similar to the effects on papillomas.

*Acknowledgements* (applicable to Papers I, II and III)

Janet Franklin and J. Rowland were responsible for treating and observing the animals, Tony Donegan cleaned up the data, Richard Gray helped with the programming, and Jini Godwin typed the manuscripts. F. Stenbäck was supported by US-PHS Grant No. CA 14710. Documented copies of the data (including the histology and onset time of each tumour) are available on magtape from R. Peto.

We are grateful to Richard Doll, Richmond T. Prehn, Fred J. Burns, Francis Roe, Sarah Parish, Peter Lee and Alice Whittemore for criticism of early drafts of these manuscripts. The figures were drawn by Cathy Harwood.

### REFERENCES

Armitage, P. & Doll, R. (1961) Stochastic models for carcinogenesis. In *Proc. 4th Berkeley Symposium on Mathematical Statistics and Probability: Biology and Problems of Health*, 4. Berkeley: University of California Press. p. 19.

Berenblum, I., Haran-Ghera, N. & Trainin, N. (1958) An experimental analysis of the "hair cycle effect" in mouse skin carcinogenesis. *Br. J. Cancer*, **12,** 402.

Berenblum, I. & Shubik, P. (1947*a*) The role of croton oil applications associated with a single painting of a carcinogen, in tumour induction of the mouse's skin. *Br. J. Cancer*, **1,** 379.

Berenblum, I. & Shubik, P. (1947*b*) A new, quantitative approach to the study of the stages of chemical carcinogenesis in the mouse's skin. *Br. J. Cancer*, **1,** 383.

Berenblum, I. & Shubik, P. (1949) The persistence of latent tumour cells induced in the mouse's skin by a single application of 9:10-dimethyl-1:2-benzanthracene. *Br. J. Cancer*, **3,** 384.

Boutwell, R. K. (1964) Some biological aspects of skin carcinogenesis. *Progr. Exp. Tum. Res.*, **4,** 207.

Hecker, E. (1971) Isolation and characterization of the co-carcinogenic principles from croton oil. In *Methods in Cancer Research*, VI. Ed. Busch. New York: Academic Press. p. 439.

Hieger, I. (1965) Studies in carcinogenesis. *Br. J. Cancer*, **19,** 761.

International Agency for Research on Cancer (1980) Guidleines for simple, sensitive significance tests for carcinogenic effects in long-term animal experiments. In *IARC Monographs on the Evaluation of the Carcinogenic Risk of Chemicals to Humans*, Suppl. 2. Ed. Montesano. Lyon: I.A.R.C. p. 311.

Manil, L., van Cantfort, J., Lapiere, C. M. & Gielen, J. E. (1981) Significant variation in mouse-skin aryl hydrocarbon hydroxylase inducibility as a function of the hair growth cycle. *Br. J. Cancer*, **43,** 210.

Peto, R. (1977) Epidemiology, multistage models and short-term mutagenicity tests. In *Origins of Human Cancer*. Ed. Hiatt *et al.* New York: Cold Spring Harbor Laboratory. p. 1403.

Peto, R. & Pike, M. C. (1973) Conservatism of the approximation $\Sigma(O-E)^2/E$ in the logrank test for survival data or tumour incidence data. *Biometrics*, **29,** 579.

Peto, R., Roe, F. J. C., Lee, P. N., Levy, L. & Clack, J. C. (1975) Cancer and ageing in mice and men. *Br. J. Cancer*, **32,** 411.

Raick, A. N. (1974) Cell proliferation and promoting action in skin carcinogenesis. *Cancer Res.*, **34,** 920.

Roe, F. J. C., Carter, R. L., Mitchley, B. C. V., Peto, R. & Hecker, E. (1972) On the persistence of tumour initiation and the acceleration of tumour progression in mouse skin tumorigenesis. *Int. J. Cancer*, **9,** 264.

Roe, F. J. C. & Salaman, M. H. (1954) A quantitative study of the power and persistence of the tumour initiating effect of ethyl carbamate (urethane) on mouse skin. *Br. J. Cancer*, **8,** 666.

Stenbäck, F. (1969) Promotion in the morphogenesis of chemically inducible skin tumours. *Acta Pathol. Microbiol. Scand.*, **77** (Suppl. 208), 325.

Stenbäck, F., Peto, R. & Shubik, P. (1981*a*) Initiation and promotion at different ages and doses in 2200 mice. II. Decrease in promotion by TPA with ageing. *Br. J. Cancer*, **44,** 15.

Stenbäck, F., Peto, R. & Shubik, P. (1981*b*) Initiation and promotion at different ages and doses in 2200 mice. III. Linear extrapolation from high doses may underestimate low-dose tumour risks. *Br. J. Cancer*, **44,** 24.

Tomatis, L., Terracini, B. & Shubik, P. (1962) Effect of a single application of croton oil in skin carcinogenesis. *Proc. Soc. Exp. Biol. Med.*, **109,** 18.

van Duuren, B. L., Orris, L. & Arroyo, E. (1963) Tumour-enhancing activity of the active principles of *Croton tiglium L. Nature*, **200,** 1115.

van Duuren, B. L., Sivak, A., Katz, C., Seidman, I. & Melchionne, S. M. (1975) The effect of aging and interval between primary and secondary treatment in two-stage carcinogenesis on mouse skin. *Cancer Res.*, **35,** 502.

van Duuren, B. L., Sivak, A. & Langsgeth, L. (1967) The tumour-promoting activity of tobacco leaf extract and whole cigarette tar. *Br. J. Cancer*, **21,** 460.

van Duuren, B. L., Smith, A. C. & Melchionne, S. M. (1978) Effect of aging in two-stage carcinogenesis on mouse skin with phorbol myristate acetate as promoting agent. *Cancer Res.*, **38,** 865.

Whittemore, A. & Keller, J. (1978) Quantitative theories of carcinogenesis. *S.I.A.M. Rev.*, **20,** 1.

### Appendix

### Table a.—*Response to promotion*

1. The cited percentages are based upon the animals which were free of all tumours when promotion started. Each percentage is a life-table estimate of the proportion that would have developed one or more tumours within 20 weeks of starting promotion.

2. The entry "T/M = x ± y in a/b/c" indicates that c animals underwent initiation, b survived to the start of promotion, and a of these survived a further 20 weeks thereafter, and that among these 20-week survivors an average of x new tumours per mouse arose during the 20 weeks after the start of promotion. y is an estimate of the standard error of x. Overall, 2200 mice were initiated, 1701 started promotion and 1082 were alive 20 weeks thereafter.

3. Finally, the crop of new tumours elicited within 20 weeks of the start of promotion is further characterized by listing in brackets, for each new tumour-bearing survivor 20 weeks after starting promotion, the number of new tumours that arose since promotion began. ("3 x 1, 2" means 3 animals had one each and one animal had 2, *etc.*)

| Protocol | Age at initiation (wks) | Ages during promotion (wks) | 300 μg initiation | 100 μg initiation | 30 μg initiation | 10 μg initiation |
|---|---|---|---|---|---|---|
| a | 8 | 11–26 | 69% T/M = 2·00 ± 0·27 in 73/80/80 (19 x 1, 9 x 2, 9 x 3, 6 x 4, 5 x 5, 6, 6, 7, 14) | 62% T/M = 1·44 ± 0·29 in 34/40/40 (9 x 1, 7 x 2, 3 x 3, 4, 6, 7) | 26% T/M = 0·66 ± 0·24 in 38/39/40 (4 x 1, 3 x 2, 3, 6, 6) | 33% T/M = 0·64 ± 0·18 in 39/40/40 (6 x 1, 4 x 2, 3, 4, 4) |
| b | 8 | 18–33 | 64% T/M = 1·25 ± 0·20 in 51/79/80 (19 x 1, 4 x 2, 6 x 3, 4, 4, 5, 6) | 25% T/M = 0·58 ± 0·19 in 33/40/40 (4 x 1, 2, 3 x 3, 4) | 39% T/M = 1·16 ± 0·32 in 38/39/40 (3 x 1, 6 x 2, 3 x 3, 5, 6, 9) | 18% T/M = 0·24 ± 0·09 in 38/40/40 (5 x 1, 2, 2) |
| c | 8 | 31–46 | 46% T/M = 0·79 ± 0·15 in 34/69/80 (10 x 1, 7 x 2, 3) | 32% T/M = 0·44 ± 0·16 in 25/37/40 (4 x 1, 2, 2, 3) | 22% T/M = 0·56 ± 0·25 in 32/37/40 (2 x 1, 3, 7) | 50% T/M = 0·80 ± 0·17 in 35/39/40 (10 x 1, 3 x 2, 4 x 3) |
| d | 8 | 51–66 | 59% T/M = 1·50 ± 0·63 in 8/31/60 (1, 1, 2, 3, 5) | 42% T/M = 0·64 ± 0·18 in 28/44/60 (6 x 1, 3 x 2, 3, 3) | 34% T/M = 0·64 ± 0·16 in 33/52/60 (7 x 1, 4 x 2, 2 x 3) | 27% T/M = 0·22 ± 0·07 in 41/56/60 (7 x 1, 2) |
| e | 8 | 71–86 | — 0/10/80 No survivors at Week 91 | 52% T/M = 0·63 ± 0·18 in 19/41/80 (6 x 1, 3 x 2) | 8% T/M = 0·16 ± 0·12 in 25/47/80 (1, 3) | 8% T/M = 0·03 ± 0·03 in 32/61/80 (1) |
| f | 48 | 51–66 | 59% T/M = 1·37 ± 0·21 in 40/59/60 (7 x 1, 10 x 2, 4 x 3, 4 x 4) | 63% T/M = 1·05 ± 0·20 in 37/58/60 (15 x 1, 6 x 2, 2 x 3, 6) | 46% T/M = 1·00 ± 0·20 in 47/59/60 (10 x 1, 8 x 2, 3, 3, 4, 5, 6) | 40% T/M = 0·63 ± 0·16 in 52/56/60 (13 x 1, 5 x 2, 4, 6) |
| g | 48 | 71–86 | 53% T/M = 0·82 ± 3·8 in 11/55/80 (3 x 1, 2,4) | 29% T/M = 0·68 ± 0·24 in 25/59/80 (5 x 1, 2, 2, 3, 5) | 31% T/M = 0·36 ± 0·12 in 36/63/80 (6 x 1, 2, 2, 3) | 15% T/M = 0·22 ± 0·13 in 36/68/80 (1, 1, 2, 4) |
| h | 68 | 71–86 | 61% T/M = 1·00 ± 0·21 in 30/78/80 (13 x 1, 4 x 2, 4, 5) | 34% T/M = 0·47 ± 0·11 in 38/78/80 (14 x 1, 2 x 2) | 21% T/M = 0·74 ± 0·33 in 38/77/80 (6 x 1, 3 x 2, 4, 12) | 10% T/M = 0·14 ± 0·07 in 36/70/80 (3 x 1, 2) |
| i | 8 | spare | 40 animals per dose-level initiated but not promoted. | | | |

TABLE b.—*The effects of age at promotion on the total tumour yield within 20 weeks of starting promotion, initiation being at a fixed age*

TABLE d.—*The effects of age at initiation on total tumour yields within 20 weeks of starting promotion, promotion being at a fixed age*

Total numbers of tumours (including second and subsequent tumours) during the 20 weeks from the start of promotion, among animals promoted at a fixed age (51–66 or 71–86 weeks, as indicated) following initiation at various previous ages, excluding animals which died less than 20 weeks after starting promotion. Abbreviations and footnote† as in Appendix Table b.

| DMBA dose | Age at promotion | Age at DMBA 8 weeks | | | Age at DMBA 48 weeks | | | Age at DMBA 68 weeks | | | Total (all ages) | | |
|---|---|---|---|---|---|---|---|---|---|---|---|---|---|
| | | S | O | E | S | O | E | S | O | E | S | O | E |
| 300 μg | 51–66 | 8 | 12 | 11·2 | 40 | 55 | 55·8 | | — | | 48 | 67 | 67·0 |
| 300 μg | 71–86 | 0 | 0 | 0·0 | 11 | 9 | 10·5 | 30 | 30 | 28·5 | 41 | 39 | 39·0 |
| 100 μg | 51–66 | 28 | 18 | 24·6 | 37 | 39 | 32·4 | | — | | 65 | 57 | 57·0 |
| 100 μg | 71–86 | 19 | 12 | 10·9 | 25 | 17 | 14·3 | 38 | 18 | 21·8 | 82 | 47 | 47·0 |
| 30 μg | 51–66 | 33 | 21 | 28·0 | 47 | 47 | 40·0 | | — | | 80 | 68 | 68·0 |
| 30 μg | 71–86 | 25 | 4 | 11·4 | 36 | 13 | 16·3 | 38 | 28 | 17·3 | 99 | 45 | 45·0 |
| 10 μg | 51–66 | 41 | 9 | 18·5 | 52 | 33 | 23·5 | | — | | 93 | 42 | 42·0 |
| 10 μg | 71–86 | 32 | 1 | 4·3 | 36 | 8 | 4·9 | 36 | 5 | 4·8 | 104 | 14 | 14·0 |
| Totals | 51–66 | 110 | 60 | 82·3 | 176 | 174 | 151·7 | | — | | 286 | 234 | 234·0 |
| (4 doses) | 71–86 | 76 | 17 | 26·6 | 108 | 47 | 46·0 | 142 | 81 | 72·4 | 326 | 145 | 145·0 |
| Total O ÷ | 51–66 | | O/E = 0·73 | | | O/E = 1·15 | | | — | | | 1·00 necessarily | |
| total E† | 71–86 | | O/E = 0·64 | | | O/E = 1·02 | | | O/E = 1·12 | | | 1·00 necessarily | |

APPENDIX TABLE e.—*The effects of age at initiation on first tumour yields at any time following promotion, promotion being at a fixed age*

| Type of lesions counted | DMBA dose (μg) | Age at promotion | Age at DMBA 8 weeks | | | Age at DMBA 48 weeks | | | Age at DMBA 68 weeks | | | Total (all ages) | | |
|---|---|---|---|---|---|---|---|---|---|---|---|---|---|---|
| | | | N | O | E | N | O | E | N | O | E | N | O | E |
| (ii) any | 300 | 51–66 | 21 | 7 | 11·3 | 58 | 32 | 27·7 | | — | | 79 | 39 | 39·0 |
| tumours | 300 | 71–86 | 8 | 0 | 1·1 | 24 | 7 | 9·6 | 78 | 32 | 28·3 | 110 | 39 | 39·0 |
| (ii) any | 100 | 51–66 | 32 | 14 | 18·3 | 58 | 32 | 27·7 | | — | | 90 | 46 | 46·0 |
| tumours | 100 | 71–86 | 36 | 14 | 8·5 | 50 | 10 | 13·8 | 72 | 17 | 18·7 | 158 | 41 | 41·0 |
| (ii) any | 30 | 51–66 | 52 | 16 | 19·7 | 59 | 26 | 22·3 | | — | | 111 | 42 | 42·0 |
| tumours | 30 | 71–86 | 46 | 3 | 8·4 | 58 | 15 | 9·0 | 77 | 12 | 12·6 | 181 | 30 | 30·0 |
| (ii) any | 10 | 51–66 | 56 | 17 | 20·2 | 56 | 23 | 19·8 | | — | | 112 | 40 | 40·0 |
| tumours | 10 | 71–86 | 60 | 6 | 5·9 | 67 | 9 | 6·8 | 70 | 5 | 7·4 | 197 | 20 | 20·0 |
| (ii) any | all* doses | 51–66 | 161 | 54 | 69·6 | 231 | 113 | 97·4 | | — | | 392 | 167 | 167·0 |
| tumours | | 71–86 | 150 | 23 | 23·9 | 199 | 41 | 39·1 | 297 | 66 | 67·0 | 646 | 130 | 130·0 |
| Total O ÷ | | 51–66 | | O/E = 0·78 | | | O/E = 1·16 | | | — | | | $P = 0{\cdot}01$ | |
| total E | | 71–86 | | O/E = 0·96 | | | O/E = 1·05 | | | O/E = 0·99 | | | $P = 0{\cdot}9$ | |
| (iii) 10 mm | all* doses | 51–66 | 182 | 12 | 17·5 | 231 | 33 | 27·5 | | — | | 413 | 45 | 45·0 |
| tumours | | 71–86 | 159 | 9 | 6·4 | 232 | 15 | 13·4 | 303 | 11 | 15·2 | 694 | 35 | 35·0 |
| Total O ÷ | | 51–66 | | O/E = 0·69 | | | O/E = 1·20 | | | — | | | $P = 0{\cdot}1$ | |
| total E | | 71–86 | | O/E = 1·40 | | | O/E = 1·12 | | | O/E = 0·72 | | | $P = 0{\cdot}1$ | |
| (iv) Malig- | all* doses | 51–66 | 180 | 7 | 10·9 | 232 | 26 | 22·1 | | — | | 412 | 33 | 33·0 |
| nancy | | 71–86 | 157 | 2 | 1·6 | 242 | 8 | 4·4 | 303 | 3 | 7·0 | 702 | 13 | 13·0 |
| Total O ÷ | | 51–66 | | O/E = 0·64 | | | O/E = 1·18 | | | — | | | $P = 0{\cdot}1$ | |
| total E | | 71–86 | | O/E = 1·27 | | | O/E = 1·81 | | | O/E = 0·43 | | | $P = 0{\cdot}1$ | |

* See footnote to Appendix Table c.